# The bradykinin B2 receptor as a context-dependent modulator of neural circuit function

**DOI:** 10.3389/fncel.2026.1830119

**Published:** 2026-05-13

**Authors:** Juliene L. S. Silva, Mariana R. Tavares, Luana Lavezo, Frederick Wasinski

**Affiliations:** Department of Neurology and Neurosurgery, Federal University of São Paulo, São Paulo, Brazil

**Keywords:** bradykinin B2 receptor (BDKRB2), dopamiergic neurons, kallikrein–kinin system, mouse model, neuroinflammantion, striatal signaling

## Abstract

The kallikrein–kinin system (KKS) is a proteolytic signaling pathway traditionally associated with vascular regulation and inflammation, but increasing evidence indicates that its components are also expressed in the central nervous system. The bradykinin B2 receptor (B2R), the main mediator of kinin signaling, has been implicated in neuronal differentiation, synaptic modulation, and activity-dependent neuroplasticity. However, its physiological role in neural circuit regulation remains poorly understood. Recent studies using cell-type–specific genetic models suggest that neuronal B2R signaling is largely dispensable for maintaining basal behavioral states. In contrast, growing evidence indicates that B2R plays a more prominent role in glial-mediated inflammatory responses, particularly in astrocytes. Here, we propose that B2R functions as a context-dependent neuromodulatory system that links neuroinflammatory signaling to neural circuit plasticity. Within this framework, B2R activation may become functionally relevant during inflammatory conditions or physiological stimulation, influencing dopaminergic circuits involved in motivation and reward-related behaviors.

## Introduction

The kallikrein–kinin system (KKS) is a proteolytic signaling cascade traditionally associated with vascular regulation, inflammation, and tissue homeostasis (Schmaier, 2016; Regoli and Barabé, 1980; Costa-Neto et al., 2008). Although kinin signaling has been extensively studied in peripheral inflammatory responses, increasing evidence indicates that components of this system are also expressed in the central nervous system (CNS). Among these components, the bradykinin B2 receptor (B2R) is constitutively expressed in neural cells and has been implicated in neuronal differentiation, synaptic modulation, and neurogenesis (Trujillo et al., 2012; Pillat et al., 2016; Kohno et al., 2008).

Beyond its role in neuronal differentiation, B2R signaling has been implicated in activity-dependent forms of neural plasticity. Experimental evidence demonstrates that B2R signaling contributes to exercise-induced hippocampal neurogenesis (Wasinski et al., 2018), suggesting that B2R may participate in adaptive forms of neural plasticity triggered by physiological stimuli.

Most studies examining the KKS in the CNS have focused on pathological conditions such as ischemic injury, traumatic brain injury, epilepsy, or neuroinflammation (Nokkari et al., 2018; Albert-Weissenberger et al., 2014). Consequently, the role of B2R signaling in physiological neural circuit regulation remains poorly understood.

Recent experimental approaches using cell-type–specific genetic models have begun to clarify the role of B2R in defined neuronal populations. Conditional deletion of B2R in tyrosine hydroxylase–expressing neurons revealed minimal alterations in locomotor activity, anxiety-like behavior, and metabolic parameters under basal physiological conditions, suggesting that dopaminergic B2R signaling is largely dispensable for maintaining basal behavioral states (Franco et al., 2024). Similarly, preliminary observations from our laboratory using conditional deletion of B2R in GABAergic neurons indicate that the absence of B2R signaling in these populations produces only limited alterations in basal behavioral phenotypes. Together, these findings suggest that neuronal B2R signaling may not be essential for maintaining baseline neural circuit function.

In contrast to the modest phenotypes observed in neuronal models, accumulating evidence indicates that B2R signaling may play a more prominent role in glial-mediated inflammatory responses. Astrocytes express functional B2R and respond to kinin stimulation by activating intracellular signaling pathways that promote inflammatory mediator production. Activation of B2R amplifies inflammatory signaling cascades, increasing the production of pro-inflammatory cytokines and nitric oxide (Nokkari et al., 2018; Dutra, 2017). Consistent with this concept, recent work from our group demonstrated that pharmacological blockade of B2R significantly attenuates LPS-induced inflammatory gene expression in primary astrocytes, supporting a key role for B2R signaling in astrocyte-driven neuroinflammation (Tavares et al., 2026). This effect was less pronounced *in vivo*, particularly in the cortex of animals with LPS-induced inflammation, suggesting that under inflammatory conditions B2R signaling may play distinct roles across brain cell types and could be more relevant in astrocytes.

## B2R signaling and basal ganglia circuits

Although B2R does not appear to play a dominant role in neuronal circuitry under basal conditions, including TH-expressing cells, its potential involvement in basal ganglia–regulated functions deserve further consideration. Experimental evidence suggests that B2R may interact with dopaminergic signaling mechanisms. Molecular studies indicate potential interactions between B2R and dopamine receptors, particularly D2 receptors, raising the possibility that inflammatory mediators may influence dopaminergic signaling within basal ganglia circuits (Niewiarowska-Sendo et al., 2017). Although the physiological relevance of this interaction remains to be fully established, these observations suggest that B2R signaling may contribute to the modulation of motivational and reward-related circuits under specific conditions. Preliminary observations from our group suggest that regionally restricted deletion of B2R within the dorsal striatum may be associated with alterations in motivational and reward-related behaviors under specific conditions (unpublished observations from our group). The dorsal striatum represents a key integrative hub within basal ganglia circuits responsible for motor control, action selection, and motivational behaviours (Gerfen and Surmeier, 2011). Dopaminergic projections from the substantia nigra pars compacta regulate the activity of medium spiny neurons, which integrate cortical inputs and shape basal ganglia output pathways.

## Discussion

### A context-dependent model of B2R function

The apparent discrepancy between minimal phenotypes observed in neuronal deletion models and the more pronounced effects observed under inflammatory or circuit-specific manipulations suggests that B2R signaling may function as a context-dependent neuromodulatory system in the brain.

One possible explanation for the limited impact of neuronal B2R deletion under basal conditions relates to the regulation of local kinin availability. Although B2R is constitutively expressed in neural cells, receptor activation depends on the local production of kinins, which are rapidly degraded by enzymes such as angiotensin-converting enzyme (ACE) and carboxypeptidases (Campbell, 2001; Hillmeister and Persson, 2012). Under basal physiological conditions, endogenous kinin levels within the brain are likely low, thereby limiting tonic B2R activation. In contrast, inflammatory or tissue injury conditions markedly increase kallikrein activity and kinin production, leading to enhanced B2R signaling. In this context, astrocytes, as key mediators of neuroimmune communication, may represent an important cellular substrate through which B2R activation influences neuronal circuit function.

In addition to their role as mediators of neuroimmune communication, astrocytes may represent a key cellular interface through which B2R signaling links inflammatory pathways to neural circuit function. Activation of B2R in astrocytes is known to engage intracellular signaling pathways, including Gq/PLC/Ca^2+^-dependent cascades, leading to activation of MAPK and NF-κB and subsequent production of pro-inflammatory mediators such as cytokines and nitric oxide (Nokkari et al., 2018; Dutra, 2017).

These signaling events may alter the synaptic microenvironment through modulation of extracellular glutamate levels, release of gliotransmitters, and cytokine-mediated effects on neuronal signaling. In this context, astrocyte-driven inflammatory responses could indirectly influence neuronal excitability and synaptic transmission within basal ganglia circuits. Although these mechanisms remain to be fully elucidated, they provide a plausible pathway linking B2R activation to circuit-level modulation.

Despite increasing interest in the role of the kallikrein–kinin system in neurological disorders, its involvement in basal ganglia circuitry remains poorly understood.

Within this framework, B2R signaling may represent a latent neuromodulatory pathway that becomes selectively engaged under defined physiological or inflammatory conditions. For example, physiological conditions such as exercise-induced plasticity and inflammatory states such as LPS-induced neuroinflammation may differentially engage B2R signaling. Together, these observations support the hypothesis that B2R functions as a molecular interface linking neuroinflammatory signaling and neural circuit plasticity in the brain. A summary of the experimental and conceptual evidence supporting this framework is presented in Table 1.

**Table 1:** Experimental evidence supporting context-dependent B2R signaling in the CNS.

Model/context	Experimental system	Key finding	Mechanistic interpretation	Relevance to hypothesis	Ref
Astrocytes (inflammatory context)	*In vitro*	B2R activation increases cytokine and NO production	Activation of proinflammatory signaling pathways (NF-κB, MAPK)	Supports glial-mediated inflammatory modulation	Nokkari et al. (2018), Dutra (2017), and Tavares et al. (2026)
LPS-induced neuroinflammation	*In vivo*	B2R blockade partially attenuates proinflammatory gene expression	Context-dependent engagement of B2R under inflammatory conditions	Supports inflammationdependent activation	Tavares et al. (2026)
TH⁺ neuronal B2R deletion	*In vivo*	Minimal behavioral and metabolic alterations	Limited role under basal conditions	Supports basal dispensability	Franco et al. (2024)
GABAergic B2R deletion	*In vivo*	Subtle behavioral changes	Cell-type–specific contribution to circuit function	Supports circuitspecific modulation	(unpublished data)
Exercise-induced plasticity	*In vivo*	B2R is required for hippocampal neurogenesis	Activity-dependent engagement of B2R signaling	Supports physiological context activation	Wasinski et al. (2018)
B2R–D2 receptor interaction	*In vitro*	B2R forms a functional complex with dopamine D2 receptor	Potential modulation of dopaminergic signaling pathways	Supports circuit-level integration	Niewiarowska-Sendo et al. (2017)

### A multi-scale mechanistic framework for B2R signaling

At the molecular level, B2R is a G protein-coupled receptor predominantly coupled to Gq, leading to activation of phospholipase C (PLC), inositol trisphosphate (IP3) production, and intracellular Ca^2+^ mobilization. This signaling cascade can engage downstream pathways such as MAPK/ERK and NF-κB, thereby regulating gene expression programs associated with inflammatory responses (Costa-Neto et al., 2008; Dutra, 2017). In astrocytes, B2R activation has been shown to promote the production of cytokines and nitric oxide, supporting its role in amplifying neuroinflammatory signaling (Nokkari et al., 2018; Tavares et al., 2026).

At the neuronal level, astrocyte activation may influence the synaptic microenvironment through modulation of extracellular glutamate clearance, release of gliotransmitters, and production of inflammatory mediators. These processes can, in turn, alter neuronal excitability and synaptic transmission.

At the circuit level, such astrocyte-mediated changes may influence dopaminergic signaling within basal ganglia circuits, particularly in the dorsal striatum, where dopaminergic inputs regulate medium spiny neuron activity. Within this framework, B2R signaling does not act as a primary driver of neuronal activity but may function as a modulatory interface that becomes engaged under specific physiological or inflammatory conditions.

B2R signaling may also influence synaptic plasticity mechanisms such as long-term potentiation (LTP) and long-term depression (LTD). Given its ability to modulate intracellular Ca^2+^ dynamics and kinase activation, B2R signaling is well positioned to influence plasticity-related pathways and glutamatergic transmission (Pillat et al., 2016; Kohno et al., 2008). However, direct evidence linking B2R signaling to canonical LTP/LTD mechanisms in basal ganglia circuits remains limited, and this represents an important area for future investigation.

These observations should be interpreted within the limitations of currently available experimental evidence and the hypothesis-driven nature of this framework.

## Conclusion

In summary, we propose that the bradykinin B2 receptor functions as a context-dependent neuromodulatory system linking neuroinflammatory signaling to neural circuit plasticity. Rather than acting as a primary determinant of basal neuronal activity, B2R signaling may become functionally relevant under conditions of physiological stimulation or inflammation. This framework highlights B2R as a potential interface between neuroimmune signaling and dopaminergic circuit modulation and provides a foundation for future experimental studies.

## Data Availability

The original contributions presented in the study are included in the article/supplementary material, further inquiries can be directed to the corresponding author/s.
